# The Meaning of Age: In a Context of Eldercare and Substance Use

**DOI:** 10.1093/geroni/igab046.873

**Published:** 2021-12-17

**Authors:** Tove Harnett, Hakan Jonson

**Affiliations:** 1 Lund University, Lund, Skane Lan, Sweden; 2 Lund University, Lund University, Skane Lan, Sweden

## Abstract

Some people age with substance abuse and social problems and several countries provide members of this population with a type of arrangement referred to as “wet” eldercare facilities. These facilities provide care for people who are judged as unable to become sober, in some cases with a lower age-limit at 50 years. The aim of this study was to investigate the meaning of age for judging the fit between the person and the arrangement. The study was based on interviews with 42 residents, 10 case workers and 21 staff members at five facilities in Sweden. Respondents were asked about the relevance of age and if the facility should include younger people as well. Some staff argued that younger people should be excluded since they could not have the history of multiple failures in treatment that was a prerequisite for admission. Regarding the low age-limit, substance abuse was said to accelerate the process of ageing so that a person aged 50 could be considered 20 years older and in need of eldercare. Residents had a tendency to equate age with activity and argued that people below the age of 50 were active and energetic and the inclusion of younger people would lead to disturbance of the calm pace of the facilities. Given that facilities have been described as “end-stations”, it was puzzling that few respondents linked the question of admitting younger person to the matter of giving up ambitions to make the person sober.

